# The Impact of Zn, Cu and Fe Chelates on the Fatty-Acid Profile and Dietary Value of Broiler-Chicken Thigh Meat

**DOI:** 10.3390/ani11113115

**Published:** 2021-10-30

**Authors:** Anna Winiarska-Mieczan, Karolina Jachimowicz, Małgorzata Kwiecień, Svitlana Kislova, Ewa Baranowska-Wójcik, Zvenyslava Zasadna, Dmytro Yanovych, Edyta Kowalczuk-Vasilev

**Affiliations:** 1 Institute of Animal Nutrition and Bromatology, University of Life Sciences in Lublin, Akademicka St. 13, 20-950 Lublin, Poland; karolina.jachimowicz@up.lublin.pl (K.J.); malgorzata.kwiecien@up.lublin.pl (M.K.); edyta.kowalczuk@up.lublin.pl (E.K.-V.); 2 State Scientific-Research Control Institute of Veterinary Medical Products and Feed Additives, 79000 Lviv, Ukraine; kislovasvit@gmail.com (S.K.); dzvinulya@gmail.com (Z.Z.); yandmyt@scivp.lviv.ua (D.Y.); 3 Department of Biotechnology, Microbiology and Human Nutrition, University of Life Sciences in Lublin, Skromna St. 8, 20-704 Lublin, Poland; ewa.baranowska@up.lublin.pl

**Keywords:** broiler chickens, glycine chelate, Zn, Cu and Fe, thigh meat, fatty acid, dietetic value

## Abstract

**Simple Summary:**

Poultry meat is deemed a product with a dietary value. The chemical composition of meat can be altered by modifying animals’ diets. Our objective was to show the impact of the addition of glycine chelates of minerals (Zn, Cu, Fe) to broiler chickens’ feed on the fatty-acid profile and dietary value of thigh meat. A positive effect was most frequently noted for Zn chelate, especially in a larger dose. The lowest levels of saturated fatty acids and atherogenic and thrombogenic indices but the highest content of polyunsaturated fatty acids n−3 and polyunsaturated fatty acids/saturated fatty acid ratios and hypocholesterolemic/hypercholesterolemic indices were noticed. The use of Cu and Fe glycine chelates was worse than Zn but did not meet the levels from the control group. These types of treatments are important in order to ensure correct functions of the body and can mitigate or even prevent the occurrence of many diseases.

**Abstract:**

This study aimed to compare the effect of Zn, Cu and Fe glycine chelates on the proximate composition, cholesterol levels, fatty-acid profile and dietary value of the thigh meat of broiler chickens. The experiment involved three hundred and fifty Ross 308 chickens divided into seven groups. The chickens were administered Zn, Cu and Fe glycine chelates in an amount corresponding to 50% of the requirement or 25% of the requirement for 42 days. It was found that the use of Zn, Cu and Fe glycine chelates did affect the fatty acid profile and dietary value of meat. A positive impact was most frequently (*p* < 0.05) noted in chickens receiving Zn chelate in an amount covering 50% of the requirement: the lowest levels of SFA and atherogenic and thrombogenic indices, the highest content of PUFA n−3 and PUFA/SFA ratios and hypocholesterolemic/hypercholesterolemic indices. Positive effects were more often recorded for chickens receiving Zn in an amount corresponding to 50% of the requirement. The results did not show that the use of Cu and Fe glycine chelates can reduce the dietary value of thigh meat in broiler chickens since, generally, the outcomes were not worse than those in the control group. It should be highlighted that due to ambiguous results, it is impossible to determine a dose of Cu and Fe glycine chelate which would be more efficient for broiler chickens. However, chickens receiving chelates in amounts corresponding to 25% of the requirement showed far better results.

## 1. Introduction

In connection with a good fatty-acid profile and low levels of cholesterol and fat, poultry meat is deemed a product with a dietary value [1]. It contains considerable amounts of polyunsaturated fatty acids (PUFA), a regular supply of which is necessary in order to ensure correct functions of the body, and significantly, it mitigates or can even prevent the occurrence of many diseases, such as coronary artery disease, myocardial infarction, autoimmune diseases and certain forms of cancer [2]. Studies have shown that the chemical composition of meat, including the content of atherogenic substances, can be altered by modifying animals’ diets. The content of PUFA and CLA (conjugated linoleic acid) isomers in poultry meat can be increased by, for instance, adding them to the feed material [3]. Another way is by adding red-ginseng expeller [4], rapeseed oil [5,6] or blueberry extract [7]. Previous studies carried out by our team showed that glycine chelates of minerals are also efficient. Additionally, it was observed that Zn chelate improved atherogenic and thrombogenic indices of poultry meat [8,9,10]. This is particularly important as cardiovascular diseases are the most common cause of mortality in Poland, accounting for about 44% of all deaths [11], and the key factor leading to such diseases, apart from unhealthy lifestyle, is excessive consumption of saturated fatty acids (SFA) and cholesterol [12], although some authors suggest that not all SFAs have an effect promoting cardiovascular diseases [13]. Exogenous fatty acids are versatile since their main characteristic is that they are incorporated in the cellular membrane, modifying its liquidity and physiological functions [13]. It is important that these changes may alter the bioavailability of eicosanoids and other lipid mediators directing cellular responses to external stimuli such as inflammations and chronic stress. Nevertheless, it is believed that the main dietary practices preventing cardiovascular diseases are the consumption of foods containing unsaturated fatty acids (UFA) and a limited consumption of SFA.

Fatty acid metabolism is regulated by Zn, Cu and Fe, among others. Zinc is not metabolised in the body but shows electrostatic interaction with anions and negatively charged groups of molecules, e.g., proteins [14]. The inclusion of zinc in a diet reduces the activity of ∆6 desaturases metabolising linoleic acid to arachidonic acid [15]. This is the essential impact of Zn on the fatty acid profile. Zinc participates in the regulation of intestinal-lipid transport and prostaglandin metabolism and in maintaining the structural and functional integrity of cellular membranes [16]. Through its insulin-mimetic and phosphodiesterase-inducing effect, Zn can regulate the release of free fatty acids from adipose tissue [17]. In contrast, an increased level of free fatty acids in blood plasma disturbs the binding of Zn^2+^ ions by albumin through an allosteric mechanism since plasma albumin binds and transports both free fatty acids and Zn^2+^ ions [18]. Copper has an influence on the systemic metabolism of lipids [19]. In the case of copper deficiency, changes are observed in the ratio of saturated to unsaturated fatty acids. The influence of Cu on the metabolism of lipid compounds in the body is manifested in the control of the expression of genes involved in the synthesis of fatty acids and cholesterol metabolism, e.g., *SREBP-1* and *SREBP-2* genes encoding sterol-regulatory element-binding protein 1 and 2, or *CYP7A1* gene-encoding cholesterol 7-alpha hydroxylase in the liver [20,21]. *SREBP-1* is involved primarily in regulating the synthesis of fatty acids, while *SREBP-2* plays an important role in modulating cholesterol biosynthesis [22]. The *SREBP-1c* isoform is the main transcription factor used by insulin to activate the gene expression of lipogenic enzymes [23]. Studies involving rats showed an increase in the level of cholesterol in the body resulting from a deficiency of Cu [24], and the use of Cu-methionine chelate in broiler chickens led to a significant decrease in the level of cholesterol in blood serum [25], while Cu-glycine chelate decreased the level of cholesterol in meat [26]. It was demonstrated that in response to a change in the level of Fe in the body, the pathways of Fe and lipids, including cholesterol, change [27]. A theory was proposed according to which ferritin, a protein that stores iron, contains binding sites modulating Fe intake and release [28]. Isothermal microcalorimetry performed by Bu et al. [28] demonstrated that arachidonic acid C:20 binds specifically with ferritin, which enhances Fe mineralisation and decreases the release of iron, thus preventing oxidation of this acid. This leads to a limiting of lipid peroxidation, oxidative damage and pro-inflammatory processes during cellular stress. A relationship between n−3 fatty acids and Fe metabolism was also confirmed, but the mechanisms remain unknown [29]. Studies involving rats receiving high doses of Fe showed a reduction in the activity of Δ5 and Δ6 desaturases, the key enzymes in the synthesis of long-chain n−6 and n−3 fatty acids [30]. In the cited study, the effect was a reduction in the level of PUFA in the liver. An overload of Fe can have an adverse effect on meat quality because Fe is a catalyst of the fat oxidation process in both raw meat and meat subject to thermal processing [31]. Moreover, Fe interacts with other minerals and especially with copper, a catalyst in oxidation reactions. This is particularly important for thigh muscles, as they contain more fat than breast muscles.

In Poland, as a standard, poultry feed is enriched with inorganic minerals, namely sulphates. However, it has been demonstrated that inorganic minerals are poorly assimilated, which leads to considerable loss of minerals with droppings and to environmental contamination [32]. Organic forms of mineral chelates with amino acids are much better assimilated [32]. In our study, Ross 308 chickens received different amounts of Zn, Cu or Fe glycine chelates. Productivity, carcass composition, bone structure and mineralisation showed positive results, and the antioxidant, dietary and organoleptic properties of the meat were corroborated [8,9,10,33,34,35]. Based on the previous studies, a decision was made to check which mineral (Cu, Zn or Fe) administered as a glycine chelate had the most efficient impact on the proximate composition, cholesterol levels, fatty acid profile and dietary value of broiler chicken thigh meat.

## 2. Materials and Methods

All the experimental procedures complied with the authorisation of the Local Ethics Committee for Animal Testing at the University of Natural Sciences in Lublin, Poland (Resolution No. 37/2011 of 17 May 2011).

### 2.1. Experimental Factor

Our previous studies showed that the coverage of the Cu, Zn, and Fe requirement of Ross 308 chickens at 50% or 25% was sufficient to obtain the desired characteristics of meat but only provided that the minerals were administered as chelates [8,9]. Therefore, in the course of the presented experiment, chickens received Cu, Zn or Fe glycine chelates in an amount corresponding to 50% or 25% of the requirement. Accurate experimental assumptions are presented in the paper by Winiarska-Mieczan et al. [35]. The productivity parameters and the antioxidant profile of thigh meat in this experiment are presented elsewhere [35]. The cited studies did not find any negative impact of Cu, Zn and Fe chelates on the production performance of chickens. However, in groups receiving Zn or Cu chelates, the meat and blood serum of birds showed a statistically higher activity of endogenous antioxidant enzymes in comparison to the group receiving chelated Fe. The use of chelated Fe led to a decrease in the antioxidant stability of meat due to increased levels of malondialdehyde (MDA). In order to increase the antioxidative stability of thigh meat, it is sufficient that broiler chickens receive Zn or Cu in the form of glycine chelate in an amount covering 25% of their requirement. However, additional tests should be performed to corroborate the advisability of using prooxidative chelated Fe in the feed of broiler chickens.

### 2.2. Birds and Experimental Design

The experiment lasted 42 days. On the first day, three hundred and fifty (350) one-day-old Ross 308 chicks were divided into seven equipotent experimental groups. In six experimental groups, the chickens received Cu, Zn or Fe glycine chelate in an amount corresponding to 50% of the requirement (experimental factor I—Table 1) or 25% of the requirement (experimental factor II—Table 2), and in the control group, Cu, Zn and Fe were added to the feed as sulphates in an amount corresponding to 100% of the requirement for each mineral. The birds were placed in cages containing 10 chicks each. The room temperature was initially 32 °C, and during the experiment, it was reduced by 2 °C every week until it reached 24 °C [35]. The birds received feed and drinking water ad libitum throughout the experiment. The requirement for Zn was determined on the basis of recommendations of producers of Ross 308 broiler chickens [36], and feed rations during the three rearing periods (starter, 1–21 days of life; grower, 22–35 days of life; finisher, 36–42 days of life) were optimised according to NRC standards [37]. The fatty-acid profile of base-feed rations is presented in Table 3.

### 2.3. Muscle Samples 

On the 42nd day of the experiment, the chickens were slaughtered. After 24 h of cooling at a temperature of 4 °C, whole thigh muscles were dissected from the carcasses, skinned and placed in plastic bags [10]. The samples were stored in a freezer at −20 °C until chemical analyses.

### 2.4. Chemical Analyses

Prior to chemical analyses, the meat was thawed at room temperature. The proximate composition of muscles and feed was determined by means of AOAC [38]: crude protein—using Kjeldahl’s method, crude ash—by Soxhlet extraction in a Velp SER 148 apparatus (Velp, Usmate, Italy), and crude ash—in a muffle furnace (550 °C, oxidant—hydrogen peroxide). Meat moisture was determined by drying the sample at 65 °C for 24 h. The fatty-acid profile was determined by gas chromatography in a Varian 3800 GC apparatus (Varian, Harfsen, the Netherlands) with an FID detector and molten silica CP-Wax 52CBWCOT using a 60 m long capillary column with internal diameter of 0.25 mm. Supelco 37 FAME Mix 47885-U (Sigma, Poznań, Poland) standard was used for analyses. The content of cholesterol was determined in an EPOLL 20 colorimeter using C3045 standard (Sigma, Bellefonte, PA, USA). The methods of determining the above-mentioned components are described in detail elsewhere [8,39]. All chemical analyses were performed in three replications.

### 2.5. Determination of pH in Meat

The pH of meat was measured 15 and 45 minutes after slaughter using a method designed by Santé and Fernandez [40] in a Testo 205 pH-meter (Testo AG, Lenzkirch, Germany). The apparatus was calibrated using certified buffer solutions with pH amounting to 4.01 and 7.0. The mean pH was calculated from three measurements of the same muscle sample.

### 2.6. Calculations and Statistical Analysis

The dietary value of meat was evaluated based on the fatty-acid profile. The following parameters were calculated: atherogenic index (AI), thrombogenic index (TI) and hypocholesterolemic to hypercholesterolemic fatty acid ratio (h/H). The parameters were calculated from the formulas [41]:

AI = (C12:0 + 4 × C14:0 + C16:0)/[∑MUFA +∑(n−6) + ∑(n−3)]; MUFA are monounsaturated fatty acids

TI = (C14:0 + C16:0 + C18:0)/[(0.5 × ∑MUFA + 0.5 × ∑(n−6) + 3 × ∑(n−3)) + (∑(n−3)/∑(n−6))] 

h/H = (C18:1 n−9 + C18:2 n−6 + C20:4 n−6 + C18:3 n−3 + C20:5 n−3 + C22:5 n−3 + C22:6 n−3)/(C14:0 + C16:0).

The content of fatty acids was also used for calculating Ʃ SFA (saturated fatty acids), Ʃ MUFA, Ʃ PUFA, Ʃ UFA, Ʃ PUFA n−3, Ʃ PUFA n−6, Ʃ PUFA/SFA, Ʃ SFA/UFA and n−6/n−3 ratio.

A statistical analysis of results was carried out using Statistica 6.0 software. One-way analysis of variance (ANOVA), by means of the t-Student-Newman-Keuls test and post hoc Tukey test, was used for calculating statistically significant differences (*p* < 0.05) between mean values for respective experimental groups, considering experimental factor I (50% of the mineral in the form of chelate) and II (25% of the mineral in the form of chelate) separately. The results were compared with those obtained in the control group shared by both experimental factors.

## 3. Results

### 3.1. Basic Chemical Composition and pH of Meat

No statistically significant impact of different levels of Zn-Gly, Cu-Gly and Fe-Gly on the content of water, crude ash, crude protein and crude fat or on the pH of broiler-chicken thigh meat was observed (Table 4).

### 3.2. Cholesterol Levels in Meat

A statistically significant impact of chelates on the total cholesterol level in meat was recorded. The meat of birds from Cu-Gly-50 and Fe-Gly-50 groups contained less (*p* < 0.05) total cholesterol than the meat of those from the control group (Table 4).

Meat from Cu-Gly-25 and Fe-Gly-25 groups contained less (*p* = 0.04) total cholesterol than meat from the control and Zn-Gly-25 groups (Table 4). 

### 3.3. Fatty Acid Profile of Meat

The use of chelates led to a significant (*p* < 0.05) alteration of the fatty-acid profile of thigh meat, but the changes were not directional (Table 5). Statistically significant differences were noted in the total fatty acids. In the Fe-Gly-50 group, more SFAs (*p* = 0.01) were found than in the Zn-Gly-50 group. A higher content (*p* = 0.01) of n−3 PUFAs was measured in the meat of chickens from the Zn-Gly-50 group compared to the Fe-Gly-50 group. In the n−3 fatty-acids family, statistically significant differences were found for α-linolenic acid (C18:3): Zn-Gly-50 > Cu-Gly-50 = Fe-Gly-50 > control and eicosatrienoic acid (C20:3): control = Zn-Gly-50 > Cu-Gly-50 = Fe-Gly-50. The level of n−6 Ʃ PUFAs was lower in the Fe-Gly-50 group in comparison to the control group (*p* = 0.01). The following relationships were noted in the n−6 fatty acid family: C18:2 acid—control > Cu-Gly-50 > Zn-Gly-50 > Fe-Gly-50; C20:2 acid—control > Zn-Gly-50 = Cu-Gly-50 > Fe-Gly-50; and C20:4 acid—Cu-Gly-50 = Fe-Gly-50 > Zn-Gly-50 = control. The highest (*p* = 0.02) n−6/n−3 ratio was observed in the control and Cu-Gly-50 groups, Ʃ PUFA/SFA ratio (*p* = 0.04) in the Zn-Gly-50 group, Cu-Gly-50 and control groups, and Ʃ SFA/UFA ratio in the Cu-Gly-50 and Fe-Gly-50 groups.

A statistically significant impact of using chelates in the Gly-25 groups on the fatty-acid profile of thigh meat was observed; however, the changes were not directional (Table 6). No statistically significant differences were noted in the total content of SFAs, MUFAs, PUFAs and UFAs. Statistically significant differences were found in the total content of n−3 PUFAs. The highest content of n−3 PUFAs was determined in the meat of chickens from the Zn-Gly-25 group, and the lowest in the control and Cu-Gly-25 groups (*p* = 0.03). For n−3 fatty acids, differences were found in the content of α-linolenic acid (C18:3): Zn-Gly-25 = Fe-Gly-25 > Cu-Gly-25 > control and of eicosatrienoic acid (C20:3): control > Cu-Gly-25 > Zn-Gly-25 = Fe-Gly-25. The highest content (*p* = 0.04) of n−6 PUFAs was found in the control, Cu-Gly-25 and Fe-Gly-25 groups. The meat of chickens from the Zn-Gly-25 group contained less (*p* = 0.04) n−6 PUFAs than in the control group. The family of n−6 fatty acids showed the following relationships: for C20:2 fatty acid—control > Zn-Gly-25 = Cu-Gly-25 > Fe-Gly-25; for C20:4 fatty acid—Cu-Gly-25 = Fe-Gly-25 > Zn-Gly-25 > control. The value of the PUFA/SFA ratio was significantly higher for Cu-Gly-25 group than for the Fe-Gly-25 group. The differences between the other groups were not significant.

### 3.4. Dietary Value of Meat

In the meat of chickens from Gly-50 groups, the value of AI was as follows: Fe-Gly-50 > Cu-Gly-50 > Zn-Gly-50 = control (Table 7). It was similar for TI: Fe-Gly-50 > Cu-Gly-50 > control > Zn-Gly-50. In comparison to the control group, the h/H ratio was not significantly different (*p* = 0.01) in the Zn-Gly-50 group, while in the Cu-Gly-50 and Fe-Gly-50 groups, this value was statistically lower.

In the Cu-Gly-25 group, the value of AI was significantly (*p* = 0.03) lower than in the Zn-Gly-25 group and insignificantly lower than in the Fe-Gly-25 and control groups (Table 7). The value of TI was as follows: Zn-Gly-25 > Fe-Gly-25 = control > Cu-Gly-25. On the other hand, the h/H ratio can be represented as: control > Cu-Gly-25 > Fe-Gly-25 > Zn-Gly-25.

## 4. Discussion

The diet of slaughter animals can modify the chemical composition of meat; for instance, it can increase the content of protein and reduce the level of fat, as demonstrated in studies involving pigs, poultry, rabbits and ruminants [42,43,44,45]. The presented study did not note any impact of chelates on the proximate composition (crude protein, total fat). This means that even in their highly assimilable form, Zn, Cu and Fe, despite their high biological significance, do not essentially regulate the synthesis of protein and fat in the body; that is, they do not increase the mass of muscles and fat. This is corroborated by results published elsewhere [35] of the carcass-composition analysis of chickens from the experiment presented there, which did not show statistically significant differences either in weight gain or in PSTM (percentage share of thigh muscles in the carcass). Additionally, the studies of Selim et al. [46] found no impact of using a Zn-methionine chelate on the percentage share of thigh and breast meat in the carcass. Furthermore, Zakaria et al. [47] and Eskandani et al. [48] did not note any impact of supplementing poultry feed with a chelate containing a complex of amino acids and Zn on the percentage share of thigh and breast meat in the carcass. Lei et al. [49] recorded inhibited accumulation of fat in the carcasses of rabbits due to the stimulating effect of Cu on lipolysis and oxidation of fatty acids, similar to studies by Skřivan et al. [50]. Our previous studies did not reveal any impact of using Cu-Gly [8] and Zn-Gly [10] on the content of fat and protein in the meat of Ross 308 broiler chickens, while the replacement of Fe sulphate with Fe glycine chelate reduced the content of fat in thigh meat; however, the protein content remained unaffected [9].

The chemical components of meat determine its quality, including sensory traits. Meat palatability is determined by its smell and taste. Smell is considered a more important characteristic because it is more easily perceived than taste. The smell of meat is, to the highest extent, determined by the SFA and UFA ratios and the content of aldehydes, ketones and alcohols [9]. The content of PUFA is particularly important as these acids are extremely sensitive to peroxidation, and volatile and non-volatile compounds produced by peroxidation are responsible for the unpleasant smell and taste of meat [51]. The ratio of Ʃ PUFA and Ʃ SFA in meat should exceed 0.45, as fat present in meat has a positive influence on the human body and, most importantly, prevents cardiovascular and chronic diseases [52]. Values below 0.45 have a hypercholesterolemic effect on humans. In the presented study, the Ʃ PUFA/SFA ratio was above 0.86 in all groups, which means that the analysed meat had a high anticholesterolemic value. The content of n−6 and n−3 PUFAs and their mutual ratio determine the hypocholesterolemic index: n−3 acids are the main regulators of the thrombogenic index, while n−6 acids are predominant in regulating the atherogenic index [53]. Meat that is healthy for humans should have low AI and TI and a high h/H index. Preferably, AI should be below 1.0, and TI should be below 0.5 [53]. In the presented studies, AI did not exceed 0.4, while TI in experimental groups ranged from 0.70 (Cu-Gly-25) to 0.79 (Fe-Gly-50), which was 40% higher than recommended. However, considering all the analysed dietary parameters, one parameter deviating from the recommendations should not affect the overall evaluation of the dietary value of thigh meat of chickens receiving glycine chelates. TI in the Control group was also higher than 0.7. The h/H ratio illustrates the effect of fatty acids on cholesterol metabolism, so the measured values should be as high as possible [53]. In the presented studies, h/H in experimental groups ranged from ca. 2.5 (Fe-Gly-50) to ca. 2.8 (Zn-Gly-50).

Poultry meat is a good source of UFA, including PUFA, the best of all terrestrial slaughter animals [10,54]. At the same time, many studies, including those carried out by our team, showed that birds’ diet can modify the fatty-acid profile and cholesterol level in poultry meat, thus affecting its dietary value [8,9,10,54]. The presented studies also found that the use of Zn, Cu and Fe glycine chelates did have an impact on the fatty-acid profile of thigh meat. Zinc (Zn), copper (Cu) and iron (Fe) have an influence on the lipid metabolism in the body through a number of mechanisms, including (1) stimulation of lipolysis, e.g., by activating lipogenic-enzyme gene expression; (2) stimulation of fatty-acid oxidation; (3) controlling expression of genes involved in the synthesis of fatty acids; (4) inhibition of lipogenesis in adipocytes; and (5) regulation of lipid transport.

The supplementation of zinc and methionine in the form of chelates (25, 50 or 100 mg/kg feed material) led to an increase in the content of SFA and decreased the level of UFA in the breast meat of broiler chickens, whereas the difference was greater for higher dosages of chelate [55]. The replacement of Zn sulphate with Zn glycine chelates (25, 50 or 100 mg/kg of feed material) altered the fatty-acid profile of breast meat, but the changes were not directional [10]. Nevertheless, the cited studies noted a clear increase in the level of n−3 and n−6 PUFAs in chickens receiving Zn chelate in comparison to those receiving Zn sulphate, but no impact of the chelate on the total content of SFA, PUFA and UFA was observed. Other studies carried out by our team showed a significant impact of Zn glycinate chelate fed to broiler chickens (25, 50 or 100 mg/kg of feed material) on the dietary value of breast meat: AI, TI and h/H. The best values were measured for Zn chelate supplemented at 50 and 25 mg/kg of feed material [10]. In the presented studies, the values of AI and TI in the meat of chickens receiving Zn chelate in an amount corresponding to 50% of the requirement were lower than in other experimental groups, and chickens from the Zn-Gly-25 group showed higher AI and TI than other experimental groups. For h/H, values higher than in other experimental groups were observed only in the meat of chickens receiving chelate covering 50% of the requirement.

Exogenous Cu can have an influence on signalling pathways associated with lipid metabolism through improved absorption, transport and utilisation of fatty acids, as shown by studies involving pigs and ruminants [56,57,58]. Additionally, it was found that the activity of some genes involved in post-absorptive lipid metabolism increased [49,56]. Copper also activates PPAR-α (peroxisome proliferator-activated receptor) and AMPK (5′AMP-activated protein kinase), which fosters a reduction in intracellular fat by stimulating lipid metabolism and inhibiting lipogenesis in adipocytes [49]. Copper plays a significant role in adipocyte metabolism, also through Cu-dependent SSAO (semicarbazide-sensitive amine oxidase), a regulator of energy processes in adipocytes. A deficiency of Cu leads to inactivation of SSAO and redirection of the metabolism to lipid-dependent pathways, which contributes to excessive growth of adipocytes and accumulation of fat [59]. Makarski et al. [60], examining turkeys fed with Cu-lysine chelate, observed a change in the fatty-acid profile: increased content of C18:1 and reduction in 14:0, 16:0 and 22:0 saturated fatty acids. In turn, the use of Cu-glycine chelates in broiler chickens had no impact on the content of SFA but did increase the levels of PUFA and n−6 PUFA in meat in comparison to chickens receiving Cu in the form of sulphate [26]. Studies by Skřivan et al. [50] showed a lower level of SFA and an increased PUFA/SFA ratio in the abdominal fat of broiler chickens receiving 200 mg Cu/kg of feed material, which could be a result of the decreased activity of 7-alpha-hydroxylase. In turn, in other studies where Cu sulphate was replaced by different amounts of Cu glycine chelate (4, 8, 16 mg/kg of feed material), no significant changes in the fatty-acid profile of breast meat were observed [8]. Similarly, in the presented study, no statistically significant effect of replacing Cu sulphate with Cu glycine chelate on the content of PUFAs, including n−3 and n−6 fatty acids, was found for any dosage of Cu (25 or 50 mg/kg of feed material). For AI and TI, in the presented studies, the results were better in chickens receiving Cu chelate in an amount corresponding to 25% of the requirement, compared to other experimental groups, than in those receiving chelate in an amount corresponding to 50% of the requirement of Cu. In contrast, the results for the h/H ratio were better in the Cu-Gly-50 group.

Previous studies carried out by our team did not show any significant impact of Fe-Gly (10, 20 or 40 mg/kg of feed material) on SFA, MUFA and PUFA totals or the n−6 to n−3 fatty acid ratio, despite differences in the content of certain fatty acids found between the groups [9]. In the presented study, the meat of chickens receiving 50 mg of chelate contained significantly less n−3 and n−6 Ʃ PUFAs, while its n−6/n−3 ratio was lower and the Ʃ SFA/UFA ratio was higher in comparison to the group receiving Fe sulphate. In contrast, in the group receiving 25 mg of chelate, only the content of n−3 Ʃ PUFA was higher than in the control group. In turn, AI and TI were better (compared to other experimental groups) in the meat of chickens receiving Fe chelate in an amount covering 25% instead of 50% of the requirement. However, h/H in the Fe-Gly-50 group was adversely lower than in other experimental groups.

For several years, our team has been investigating the impact of feed ingredients on the quality of poultry and swine meat. We have also been analysing the effect of supplementation with chelated minerals on the quality of animal meat. However, each of the chelates has always been examined separately, so their effectiveness has never been compared. We were only aware that each of them had a specific effect on the tested parameters in comparison to sulphates but that these effects differed. For instance, it was noted in earlier studies that both Cu chelate [8] and Fe chelate [9] induced a statistically significant decrease in the cholesterol content in meat, but the present study indicated that their effects were equally strong (no statistically significant differences were found at *p* < 0.05) in contrast to Zn. In the case of the fatty-acid profile in the meat, it was found that the results in the Zn-chelate groups were generally more favourable than in the Fe-chelate groups, especially in the Gly-50 groups. The available literature reports describe the use of chelates of one mineral component only. We believe that the effectiveness of chelates in the nutrition of different animal species with different production purposes (meat, eggs, milk) should be compared; otherwise, it is impossible to indicate a product with the best effect. In the case of our study, the comparison of the effectiveness of chelates of various minerals (Cu, Zn, and Fe) will enable livestock farmers to choose the most beneficial chelate that can be used in poultry feeding without incurring unnecessary costs. Mineral chelates intended for poultry nutrition are 2–3 times more expensive than sulphates in Poland, but chelates are more easily digestible. Meat offered to consumers must meet certain standards. The most important are its dietary value (determined by the fatty-acid profile) and organoleptic quality. Since chelates are likely to be massively introduced into feed mixtures for broiler chickens in the near future, a potential reduction of the nutritional value and quality of poultry meat caused by these additives should be assessed. Food safety should be the primary goal of food producers.

## 5. Conclusions

To sum up, the presented studies found that the use of Zn, Cu and Fe glycine chelates did have an impact on the dietary value of meat. Analysing the results, a positive effect was most frequently (*p* < 0.05) noted for Zn chelate in an amount covering 50 % of the requirement: the lowest level of SFA, AI and TI, and the highest of n−3 PUFA and PUFA/SFA and h/H. This means that to ensure a high dietary value of meat, Zn glycine chelate should be administered to broiler chickens in an amount covering 50% of the requirement, which, at the same time, ensures high antioxidant stability of meat, as described elsewhere [35]. However, the results did not show that the use of Cu and Fe glycine chelates reduce the dietary value of thigh meat in broiler chickens, since generally, the outcomes were not worse than those in the control group. It should be highlighted that, due to ambiguous results, it is impossible to determine a dose of Cu and Fe glycine chelate which would be more efficient for broiler chickens. However, chickens receiving chelates in amounts corresponding to 25% of the requirement showed far better results.

## Figures and Tables

**Table 1:** Experimental design—treatment I (50% of the mineral in the form of chelate).

Feeding Groups				
	Control	Zn-Gly-50	Cu-Gly-50	Fe-Gly-50
Starter (1–21 days)	Standard mixture ^a,b^ (contained 99.71 mg Zn, 22.10 mg Cu and 42.31 mg Fe per kg at the form of sulphates) *	Standard mixture (contained 63.07 mg Zn per kg at the form of glycine chelate) **	Standard mixture (contained 11.78 mg Cu per kg at the form of glycine chelate) **	Standard mixture (contained 22.03 mg Fe per kg at the form of glycine chelate) **
Grower (22–35 days)	Standard mixture (contained 98.50 mg Zn, 22.21 mg Cu and 39.82 mg Fe per kg at the form of sulphates) *	Standard mixture (contained 56.92 mg Zn per kg at the form of glycine chelate) **	Standard mixture (contained 13.15 mg Cu per kg at the form of glycine chelate) **	Standard mixture (contained 25.30 mg Fe per kg at the form of glycine chelate) **
Finisher (36–42 days)	Standard mixture (contained 98.52 mg Zn, 21.95 mg Cu and 38.61 mg Fe per kg at the form of sulphates) *	Standard mixture (contained 56.09 mg Zn per kg at the form of glycine chelate) **	Standard mixture (contained 12.02 mg Fe per kg at the form of glycine chelate) **	Standard mixture (contained 20.46 mg Fe per kg at the form of glycine chelate) **
Access to feed and water	Free	Free	Free	Free
Number of chickens in the experiments	50	50	50	50
Number of chickens for dissection	10	10	10	10

^a^ Composition of the standard mixtures: maize, wheat, soybean meal 46%, soybean oil, monocalcium phosphate, limestone, sodium bicarbonate, NaCl, vitamin—mineral premix, fat-protein concentrate, DL-methionine 99%, L-lysine HCl, L-threonine 99%; * at 100% recommended levels for Ross broiler chicks [36]; ^b^ nutrient composition of basal diet: starter (1–21 days)—energy 12.7 MJ kg^−1^, crude protein 20.2%, crude fibre 3.06%, crude fat 4.66%, lysine 1.29%, methionone + cysteine 0.93%, grower (22–35 days)—energy 13.1 MJ kg^−1^, crude protein 18.2%, crude fibre 2.99%, crude fat 6.08%, lysine 1.13%, methionone + cysteine 0.83, finisher (36–42 days)—energy 13.2 MJ kg^−1^, crude protein 18.1%, crude fibre 2.99%, crude fat 6.43%, lysine 1.09%, methionone + cysteine 0.81; ** at 50% recommendation levels for Ross broiler chicks [36].

**Table 2:** Experimental design—treatment II (25% of the mineral in the form of chelate).

Feeding Groups				
	Control	Zn-Gly-25	Cu-Gly-25	Fe-Gly-25
Starter (1–21 days)	Standard mixture ^a,b^ (contained 99.71 mg Zn, 22.10 mg Cu and 42.31 mg Fe per kg at the form of sulphates) *	Standard mixture (contained 27.03 mg Zn per kg at the form of glycine chelate) **	Standard mixture (contained 6.12 mg Cu per kg at the form of glycine chelate) **	Standard mixture (contained 13.01 mg Fe per kg at the form of glycine chelate) **
Grower (22–35 days)	Standard mixture (contained 98.50 mg Zn, 22.21 mg Cu and 39.82 mg Fe per kg at the form of sulphates) *	Standard mixture (contained 34.23 mg Zn per kg at the form of glycine chelate) **	Standard mixture (contained 6.97 mg Cu per kg at the form of glycine chelate) **	Standard mixture (contained 11.83 mg Fe per kg at the form of glycine chelate) **
Finisher (36–42 days)	Standard mixture (contained 98.52 mg Zn, 21.95 mg Cu and 38.61 mg Fe per kg at the form of sulphates) *	Standard mixture (contained 30.05 mg Zn per kg at the form of glycine chelate) **	Standard mixture (contained 6.70 mg Fe per kg at the form of glycine chelate) **	Standard mixture (contained 12.40 mg Fe per kg at the form of glycine chelate) **
Access to feed and water	Free	Free	Free	Free
Number of chickens in the experiments	50	50	50	50
Number of chickens for dissection	10	10	10	10

^a^ Composition of the standard mixtures: maize, wheat, soybean meal 46%, soybean oil, monocalcium phosphate, limestone, sodium bicarbonate, NaCl, vitamin—mineral premix, fat-protein concentrate, DL-methionine 99%, L-lysine HCl, L-threonine 99%; * at 100 % recommendation levels for Ross broiler chicks [36]; ^b^ nutrient composition of basal diet: starter (1–21 days)—energy 12.7 MJ kg^−1^, crude protein 20.2%, crude fibre 3.06%, crude fat 4.66%, lysine 1.29%, methionone + cysteine 0.93%, grower (22–35 days)—energy 13.1 MJ kg^−1^, crude protein 18.2%, crude fibre 2.99%, crude fat 6.08%, lysine 1.13%, methionone + cysteine 0.83, finisher (36–42 days)—energy 13.2 MJ kg^−1^, crude protein 18.1%, crude fibre 2.99%, crude fat 6.43%, lysine 1.09%, methionone + cysteine 0.81; ** at 25% recommendation levels for Ross broiler chicks [36].

**Table 3:** Main fatty-acid profile of the basal mixtures, g/100 g.

	Starter 1–21 Days	Grower 22–35 Days	Finisher 36–42 Days
Myristic (14:0)	0.02	0.08	0.07
Palmitic (16:0)	1.39	1.19	1.10
Stearic (18:0)	0.31	0.29	0.35
Oleic (18:1n-9)	2.24	2.20	2.16
Linoleic (18:2n-6)	4.69	4.97	4.92
Linolenic (18:3n-3)	1.16	0.87	0.91

**Table 4:** The meat pH and chemical composition of thigh-meat samples.

**Treatment I—50% of the Mineral in the Form of Chelate**						
	**Control**	**Zn-Gly-50**	**Cu-Gly-50**	**Fe-Gly-50**	**SEM**	***p* Value**
pH_15_	6.15 ± 0.05	6.21 ± 0.03	6.20 ± 0.05	6.17 ± 0.01	1.33	0.14
pH_45_	5.32 ± 0.03	5.35 ± 0.04	5.24 ± 0.05	5.31 ± 0.06	1.20	0.33
Moisture, %	73.0 ± 0.83	72.6 ± 0.61	73.9 ± 0.99	73.7 ± 1.15	5.88	0.29
Crude ash, %	1.20 ± 0.02	1.16 ± 0.01	1.13 ± 0.01	1.09 ± 0.07	2.85	0.08
Crude protein, %	18.9 ± 0.74	19.1 ± 0.88	19.6 ± 1.02	19.6 ± 0.61	10.3	0.10
Crude fat, %	6.77 ± 1.38	6.55 ± 1.03	6.62 ± 0.95	6.56 ± 1.19	5.56	0.25
Cholesterol, mg/100 g	89.7 ± 5.33 ^b^	88.3 ± 6.41 ^b^	79.2 ± 5.09 ^a^	81.0 ± 4.77 ^a^	1.35	0.03
**Treatment II—25% of the Mineral in the Form of Chelate**						
	**Control**	**Zn-Gly-25**	**Cu-Gly-25**	**Fe-Gly-25**	**SEM**	***p* Value**
pH_15_	6.22 ± 0.07	6.17 ± 0.03	6.24 ± 0.07	6.20 ± 0.04	1.23	0.09
pH_45_	5.51 ± 0.04	5.55 ± 0.02	5.43 ± 0.05	5.47 ± 0.02	1.54	0.10
Moisture, %	73.3 ± 1.11	73.2 ± 1.37	73.5 ± 1.42	73.6 ± 1.23	12.1	0.08
Crude ash, %	1.00 ± 0.01	1.00 ± 0.01	0.99 ± 0.01	1.00 ± 0.01	2.33	0.22
Crude protein, %	18.9 ± 0.30	19.2 ± 0.20	19.2 ± 0.27	18.8 ± 0.18	7.24	0.06
Crude fat, %	6.60 ± 0.66	6.67 ± 0.59	6.69 ± 0.14	6.72 ± 0.30	3.38	0.06
Cholesterol, mg/100 g	91.3 ± 4.55 ^b^	88.2 ± 6.32 ^b^	80.3 ± 3.98 ^a^	82.5 ± 3.76 ^a^	2.76	0.04

^a, b^—means with different superscripts in lines differ at *p* < 0.05; SEM—standard error of the means; pH—potential of hydrogen.

**Table 5:** Fatty acid profile (g/100 g of total fatty acids) and dietetic values of thigh meat samples—treatment I (50% of the mineral in the form of chelate).

	Control	Zn-Gly-50	Cu-Gly-50	Fe-Gly-50	SEM	*p* Value
6:0	0.012 ± 0.01 ^b^	0.010 ± 0.01 ^a,b^	0.011 ± 0.01 ^a,b^	0.009 ± 0.01 ^a^	0.33	<0.01
8:0	0.012 ± 0.01 ^a^	0.015 ± 0.01 ^b^	0.018 ± 0.02 ^c^	0.022 ± 0.01 ^d^	0.17	<0.01
10:0	0.014 ± 0.01 ^b^	0.010 ± 0.01 ^a^	0.011 ± 0.01 ^a^	0.016 ± 0.01 ^c^	0.54	0.01
12:0	0.254 ± 0.02 ^b^	0.231 ± 0.03 ^a^	0.249 ± 0.02 ^b^	0.233 ± 0.02 ^a^	1.74	0.04
14:0	0.434 ± 0.16 ^a^	0.516 ± 0.04 ^d^	0.470 ± 0.07 ^b^	0.498 ± 0.09 ^c^	1.45	0.02
15:0	0.103 ± 0.03 ^a^	0.111 ± 0.02 ^b^	0.099 ± 0.01 ^a^	0.103 ± 0.01 ^a^	0.87	0.03
16:0	22.16 ± 2.39 ^a^	22.13 ± 2.25 ^a^	23.10 ± 2.43 ^b^	23.82 ± 2.58 ^c^	5.33	0.05
17:0	0.149 ± 0.02 ^c^	0.133 ± 0.02 ^a^	0.130 ± 0.02 ^a^	0.141 ± 0.02 ^b^	0.47	0.01
18:0	6.501 ± 0.69 ^a^	6.486 ± 0.57 ^a^	6.974 ± 1.31 ^b^	6.394 ± 1.12 ^b^	0.88	0.03
20:0	0.121 ± 0.03 ^b^	0.115 ± 0.02 ^a^	0.135 ± 0.02 ^c^	0.131 ± 0.01 ^c^	0.12	0.03
16:1	2.671 ± 0.53 ^b^	2.429 ± 0.79 ^a^	3.134 ± 0.62 ^c^	3.340 ± 0.33 ^d^	0.55	<0.01
17:1	0.049 ± 0.03 ^c^	0.055 ± 0.02 ^c^	0.030 ± 0.02 ^b^	0.025 ± 0.01 ^a^	0.04	<0.01
18:1 n-9	34.48 ± 1.57 ^c^	35.16 ± 1.45 ^d^	32.87 ± 1.67 ^a^	33.20 ± 1.31 ^b^	9.31	0.03
18:1 n-11	2.453 ± 0.16 ^c^	2.264 ± 0.42 ^b^	2.110 ± 0.28 ^a^	2.511 ± 0.21 ^d^	0.48	0.03
20:1 n-7	0.068 ± 0.02 ^b^	0.061 ± 0.01 ^a^	0.070 ± 0.01 ^b^	0.063 ± 0.01 ^a^	0.02	0.04
20:1 n-9	0.015 ± 0.01 ^a,b^	0.020 ± 0.01 ^b^	0.013 ± 0.01 ^a^	0.018 ± 0.01 ^b^	0.10	0.02
20:1 n-11	0.300 ± 0.05 ^b^	0.295 ± 0.06 ^b^	0.254 ± 0.09 ^a^	0.305 ± 0.06 ^b^	0.11	<0.01
18:2 n-6	25.53 ± 1.47 ^d^	24.70 ± 1.68 ^b^	25.16 ± 1.12 ^c^	24.11 ± 2.63 ^a^	4.55	0.03
20:2 n-6	0.319 ± 0.05 ^c^	0.293 ± 0.07 ^b^	0.284 ± 0.03 ^b^	0.220 ± 0.08 ^a^	0.34	0.02
18:3 n-3	2.298 ± 0.17 ^a^	2.650 ± 0.35 ^c^	2.499 ± 0.18 ^b^	2.474 ± 0.24 ^b^	0.56	0.02
20:3 n-3	0.174 ± 0.04 ^b^	0.174 ± 0.01 ^b^	0.148 ± 0.02 ^a^	0.140 ± 0.02 ^a^	0.15	0.01
20:4 n-6	0.101 ± 0.01 ^a^	0.100 ± 0.01 ^a^	0.115 ± 0.02 ^b^	0.116 ± 0.02 ^b^	0.07	0.01
Σ SFA	30.23 ± 3.11 ^a,b^	29.77 ± 2.30 ^a^	30.71 ± 2.07 ^a,b^	31.94 ± 3.56 ^b^	6.22	0.01
Σ MUFA	39.99 ± 1.64	40.23 ± 1.51	38.45 ± 1.92	39.43 ± 1.48	8.74	0.07
Σ PUFA	28.42 ± 1.55	27.92 ± 1.65	28.21 ± 1.12	27.06 ± 2.67	3.09	0.06
Σ UFA	68.41 ± 2.49	68.15 ± 2.12	66.65 ± 2.28	66.49 ± 3.36	12.8	0.05
Σ PUFA n-3	2.471 ± 0.20 ^a,b^	2.824 ± 0.35 ^b^	2.646 ± 0.19 ^a,b^	2.614 ± 0.24 ^a^	0.98	0.01
Σ PUFA n-6	25.95 ± 1.47 ^b^	25.09 ± 1.66 ^a,b^	25.56 ± 1.11 ^a,b^	24.44 ± 2.65 ^a^	3.22	0.01
Σ PUFA/SFA	0.952 ± 0.14 ^b^	0.945 ± 0.12 ^b^	0.922 ± 0.07 ^a,b^	0.864 ± 0.18 ^a^	0.27	0.04
Σ SFA/UFA	0.434 ± 0.06 ^a^	0.429 ± 0.05 ^a^	0.452 ± 0.05 ^b^	0.474 ± 0.08 ^b^	0.65	0.03
n-6/n-3	10.54 ± 0.87 ^b^	9.011 ± 1.30 ^a^	9.705 ± 0.86 ^a,b^	9.415 ± 1.29 ^a^	4.09	0.02

^a, b, c, d^—means with different superscripts in lines differ at *p* < 0.05; SEM—standard error of the means; SFA—saturated fatty acids; MUFA—monounsaturated fatty acids; PUFA—polyunsaturated fatty acids; UFA—unsaturated fatty acids.

**Table 6:** Fatty acid profile (g/100 g of total fatty acids) and dietetic values of thigh meat samples—treatment II (25% of the mineral in the form of chelate).

	Control	Zn-Gly-25	Cu-Gly-25	Fe-Gly-25	SEM	*p* Value
6:0	0.012 ± 0.01 ^a^	0.026 ± 0.01 ^b^	0.028 ± 0.03 ^b,c^	0.034 ± 0.02 ^c^	0.02	<0.01
8:0	0.012 ± 0.01 ^a,b^	0.011 ± 0.01 ^a^	0.014 ± 0.01 ^b^	0.021 ± 0.01 ^c^	0.03	<0.01
10:0	0.014 ± 0.01 ^a^	0.014 ± 0.01 ^a^	0.018 ± 0.01 ^b^	0.021 ± 0.01 ^c^	0.03	<0.01
12:0	0.254 ± 0.02 ^c^	0.216 ± 0.02 ^a,b^	0.204 ± 0.07 ^a^	0.224 ± 0.06 ^b^	0.11	0.03
14:0	0.434 ± 0.16 ^c^	0.393 ± 0.15 ^a^	0.395 ± 0.10 ^a^	0.414 ± 0.09 ^b^	0.10	0.03
15:0	0.103 ± 0.03 ^a^	0.102 ± 0.02 ^a^	0.108 ± 0.02 ^a^	0.118 ± 0.01 ^b^	0.03	0.01
16:0	22.16 ± 2.39	22.97 ± 1.86	22.40 ± 1.63	22.70 ± 0.85	3.98	0.02
17:0	0.149 ± 0.02 ^a^	0.154 ± 0.01 ^a^	0.154 ± 0.01 ^a^	1.168 ± 0.04 ^b^	0.95	0.01
18:0	6.501 ± 0.69 ^b^	6.379 ± 1.27 ^a^	6.469 ± 0.60 ^c^	6.475 ± 0.63 ^d^	1.34	0.02
20:0	0.121 ± 0.03 ^a^	0.130 ± 0.02 ^b^	0.151 ± 0.07 ^c^	0.160 ± 0.02 ^c^	0.33	0.04
16:1	2.671 ± 0.53 ^a^	3.293 ± 0.33 ^c^	3.103 ± 0.17 ^b^	3.195 ± 0.46 ^b,c^	0.79	<0.01
17:1	0.049 ± 0.03 ^c^	0.033 ± 0.02 ^b^	0.024 ± 0.01 ^a^	0.022 ± 0.01 ^a^	0.05	<0.01
18:1 n-9	34.48 ± 1.57	34.43 ± 1.73	35.40 ± 0.66	34.65 ± 1.12	6.34	0.06
18:1 n-11	2.453 ± 0.16 ^b^	2.274 ± 0.19 ^a^	2.232 ± 0.23 ^a^	2.235 ± 0.14 ^a^	1.08	0.01
20:1 n-7	0.068 ± 0.02 ^c^	0.050 ± 0.02 ^b^	0.040 ± 0.01 ^a^	0.041 ± 0.01 ^a^	0.11	0.01
20:1 n-9	0.015 ± 0.01 ^a^	0.023 ± 0.01 ^b^	0.020 ± 0.01 ^b^	0.019 ± 0.01 ^b^	0.13	0.02
20:1 n-11	0.300 ± 0.05 ^b^	0.257 ± 0.05 ^a^	0.246 ± 0.05 ^a^	0.252 ± 0.06 ^a^	0.54	0.01
18:2 n-6	25.53 ± 1.47	24.61 ± 1.26	25.16 ± 1.00	24.73 ± 0.78	4.45	0.05
20:2 n-6	0.319 ± 0.05 ^c^	0.196 ± 0.04 ^b^	0.210 ± 0.11 ^b^	0.159 ± 0.09 ^a^	0.33	0.01
18:3 n-3	2.298 ± 0.17 ^a^	2.419 ± 0.29 ^c^	2.355 ± 0.18 ^b^	2.408 ± 0.13 ^c^	1.68	0.01
20:3 n-3	0.174 ± 0.04 ^c^	0.147 ± 0.03 ^a^	0.161 ± 0.02 ^b^	0.148 ± 0.02 ^a^	0.22	0.02
20:4 n-6	0.101 ± 0.01 ^a^	0.113 ± 0.03 ^b^	0.166 ± 0.03 ^c^	0.158 ± 0.02 ^c^	0.40	<0.01
Σ SFA	30.23 ± 3.11	30.54 ± 1.58	29.19 ± 2.61	31.32 ± 0.97	5.07	0.06
Σ MUFA	39.99 ± 1.64	40.32 ± 1.60	41.04 ± 0.75	40.39 ± 0.89	10.8	0.05
Σ PUFA	28.42 ± 1.55	27.48 ± 1.45	28.05 ± 0.98	27.60 ± 0.79	7.12	0.07
Σ UFA	68.41 ± 2.49	67.81 ± 2.38	69.09 ± 1.11	68.00 ± 0.85	9.84	0.05
Σ PUFA n-3	2.471 ± 0.20 ^a^	2.566 ± 0.29 ^c^	2.516 ± 0.17 ^a,b^	2.555 ± 0.14 ^b^	0.32	0.03
Σ PUFA n-6	25.95 ± 1.47 ^b^	24.92 ± 1.29 ^a^	25.53 ± 1.11 ^a,b^	25.05 ± 0.77 ^a,b^	7.01	0.04
Σ PUFA/SFA	0.952 ± 0.14 ^a,b^	0.903 ± 0.08 ^a,b^	0.970 ± 0.12 ^b^	0.888 ± 0.04 ^a^	0.32	0.03
Σ SFA/UFA	0.434 ± 0.06	0.441 ± 0.03	0.413 ± 0.04	0.435 ± 0.02	0.21	0.07
n-6/n-3	10.54 ± 0.87	9.797 ± 0.96	10.21 ± 1.07	9.827 ± 0.59	1.09	0.08

^a, b, c, d^—means with different superscripts in lines differ at *p* < 0.05; SEM—standard error of the means; SFA—saturated fatty acids; MUFA—monounsaturated fatty acids; PUFA—polyunsaturated fatty acids; UFA—unsaturated fatty acids.

**Table 7:** Dietetic values of thigh-meat samples.

**Treatment I—50% of the Mineral in the Form of Chelate**						
	**Control**	**Zn-Gly-50**	**Cu-Gly-50**	**Fe-Gly-50**	**SEM**	***p* Value**
AI	0.354 ^a^	0.360 ^a^	0.380 ^b^	0.394 ^c^	1.09	0.04
TI	0.733 ^b^	0.709 ^a^	0.752 ^c^	0.790 ^d^	0.36	0.03
h/H	2.798 ^c^	2.795 ^c^	2.599 ^b^	2.498 ^a^	0.55	0.01
**Treatment II—25% of the Mineral in the Form of Chelate**						
	**Control**	**Zn-Gly-25**	**Cu-Gly-25**	**Fe-Gly-25**	**SEM**	***p* Value**
AI	0.354 ^a,b^	0.363 ^b^	0.348 ^a^	0.359 ^a,b^	2.34	0.03
TI	0.733 ^b^	0.741 ^c^	0.697 ^a^	0.731 ^b^	0.70	0.03
h/H	2.798 ^d^	2.657 ^a^	2.782 ^c^	2.684 ^b^	1.05	0.02

^a, b, c, d^—means with different superscripts in lines differ at *p* < 0.05; SEM—standard error of the means; AI—atherogenic indices; TI—thrombogenic indices; h/H—hypocholesterolemic/hypercholesterolemic ratio.

## Data Availability

The data presented in this study are available on request from the corresponding author.
